# Genetic diversity in a unique population of dugong (*Dugong dugon*) along the sea coasts of Thailand

**DOI:** 10.1038/s41598-021-90947-4

**Published:** 2021-06-02

**Authors:** Anocha Poommouang, Wannapimol Kriangwanich, Kittisak Buddhachat, Janine L. Brown, Promporn Piboon, Siriwadee Chomdej, Jatupol Kampuansai, Supamit Mekchay, Patcharaporn Kaewmong, Kongkiat Kittiwattanawong, Korakot Nganvongpanit

**Affiliations:** 1 Animal Bone and Joint Research Laboratory, Department of Veterinary Biosciences and Public Health, Faculty of Veterinary Medicine, Chiang Mai University, Chiang Mai, 50100 Thailand; 2 Excellence Center in Veterinary Bioscience, Chiang Mai, 50100 Thailand; 3 Department of Biology, Faculty of Science, Naresuan University, Phitsanulok, 65000 Thailand; 4 Smithsonian Conservation Biology Institute, Center for Species Survival, 1500 Remount Road, Front Royal, VA 22630 USA; 5 Department of Biology, Faculty of Science, Chiang Mai University, Chiang Mai, 50200 Thailand; 6 Department of Animal and Aquatic Sciences, Faculty of Agriculture, Chiang Mai University, Chiang Mai, 50200 Thailand; 7 Phuket Marine Biological Center, Phuket, 83000 Thailand

**Keywords:** Marine biology, Haplotypes, Population genetics

## Abstract

Dugong (*Dugong dugon*) populations have been shrinking globally, due in large part to habitat fragmentation, degradation and ocean pollution, and today are listed as Vulnerable by the IUCN. Thus, determining genetic diversity in the remaining populations is essential for conservation planning and protection. In this study, measures of inter-simple sequence repeat (ISSR) markers and mtDNA D-loop typing were used to evaluate the genetic diversity of 118 dugongs from skin samples of deceased dugongs collected in Thai waters over a 29-year period. Thirteen ISSR primers revealed that dugongs from the Andaman Sea and Gulf of Thailand exhibited more genetic variation in the first 12 years of the study (1990–2002) compared to the last decade (2009–2019). Dugongs from the Andaman Sea, Trang, Satun and some areas of Krabi province exhibited greater diversity compared to other coastal regions of Thailand. Eleven haplotypes were identified, and when compared to other parts of the world (235 sequences obtained from NCBI), five clades were apparent from a total 353 sequences. Moreover, dugongs from the Andaman Sea were genetically distinct, with a separate haplotype belonging to two clades found only in Thai waters that separated from other groups around 1.2 million years ago. Genetic diversity of dugongs in present times was less than that of past decades, likely due to increased population fragmentation. Because dugongs are difficult to keep and breed in captivity, improved in situ conservation actions are needed to sustain genetically healthy wild populations, and in particular, the specific genetic group found only in the Andaman Sea.

## Introduction

The dugong (*Dugong dugon*) is one of four species belonging to the order Sirenia, Family *Dugongidae*. It is distinct compared to other marine mammals in being entirely herbivorous, and as such plays an important role in maintaining coastal ecosystems. Dugongs are critically endangered, the IUCN has listed dugongs as vulnerable to extinction on a global scale because numbers have declined by at least 20% over the last 90 years^1^, and will be endangered unless circumstances threatening their survival are alleviated and reproduction is improved^2,3^. In addition, it is listed in Appendix I of the Convention on International Trade in Endangered Species^4^. Dugong populations have declined drastically since the 1960s throughout much of their natural range. For example, in Australia, the estimated rate of decline averaged about 8.7% per year between 1962 and 1999, resulting in a 97% reduction in initial catch rates over a 38-year period^1^. The largest populations today are found along the coasts of Australia (10,000 dugongs)^5^, followed by the Arabian/Persian Gulf (6000)^6,7^, Red Sea (2000)^8^, New Caledonia (898)^5,9^, and Mozambique (300)^8^. Dugongs have completely disappeared from areas in Japan (Sakishima Shoto islands), Hong Kong, Maldives, Mauritius, Philippines, Taiwan, Cambodia, and Vietnam^1^.

In Thailand, dugongs are categorized as a rare marine mammal by the Wild Animal Reservation and Protection Act, B.E.2553^10^. As in other regions, vulnerability is mainly caused by habitat loss, particularly destruction of seagrasses. The natural distribution of dugongs in Thailand includes coastal areas along the west and east sides of the Gulf of Thailand (Chonburi, Rayong, Prachuap Khiri Khan, Chumphon, and Surat Thani provinces) and Andaman Sea (Ranong, Phang Nga, Phuket, Krabi, Trang, and Satun provinces). The estimated number of dugongs in Thailand was less than 200 in 2017, with the majority (150–170) living in the Hat Chao Mai National Marine Park and Mu Ko Libong, both non-hunting areas in Trang province where seagrass is still intact^11^. In Thailand, 24 strandings were reported between October 1, 2018–September 30, 2019, most of which (56%) were due to human activities, including entanglement in fishing equipment and boat strikes^12^. A previous study over a 33-year period (1962–2008) recorded a total of 282 dugong strandings in Thailand^13^. For most of those, cause of death was unknown, but others were due to gillnets, stationary traps, fishing gear, boat strikes, or shark attacks. Increased long-term population monitoring and mitigating actions to protect animals and seagrass habitats are needed to conserve global dugong populations.

Population substructure has important implications for a species’ ecology and evolution. As such, knowledge of structuring is critical for the conservation and management of natural populations. In the past decades, researchers have studied genetic diversity of Sirenia in several habitats, for instance, Australia and the Indian Ocean using mitochondrial DNA (mtDNA) amplification of overlapping fragments of the D-loop region^3,14^. In a previous study, Plon et al.^3^ found a 355 bp sequence in the mtDNA that matched dugongs from Australia and Indonesia, but revealed several new and divergent mtDNA lineages in the Indian Ocean. Using nuclear DNA (nDNA) microsatellite markers, a study of dugongs in Australia found that the genetic diversity was low. A significant population structure was detected and mean pairwise relatedness values within populations were low as well^14^. Gene diversity and population structure in dugongs in Thailand have been assessed by analyses of D-loop mitochondrial DNA region sequences, cytochrome c oxidase subunit I (COI), and autosomal microsatellites^11,15^. Low genetic distance was observed between dugong populations in the Andaman Sea and Gulf of Thailand, which suggests that gene flow between populations might be occurring^11^. Moreover, the three mtDNA haplogroups discovered in dugongs of Thai waters were not differentiated by region. Microsatellite analyses provided a signal of dispersal between the Andaman Sea and Gulf of Thailand indicating genetic variation has remained higher than expected given the declining numbers of dugongs in each region^15^.

A major limitation to genetic studies of dugongs has been low sample numbers. This study took advantage of 29 years’ worth of banked skin samples from stranded dugongs off both coasts of Thailand to assess genetic diversity over time and between those two regions. We also determined how diversity and phylogeographphic structuring compares between dugongs in Thai waters to those globally based on sequences available in the National Center for Biotechnology Information (NCBI) Genbank^®^. The results of this study provide important information on genetic diversity and phylogeographphic structuring of dugongs in Thailand and for understanding the distribution of populations according to world geography.

## Materials and methods

### Samples

**Figure 1:**
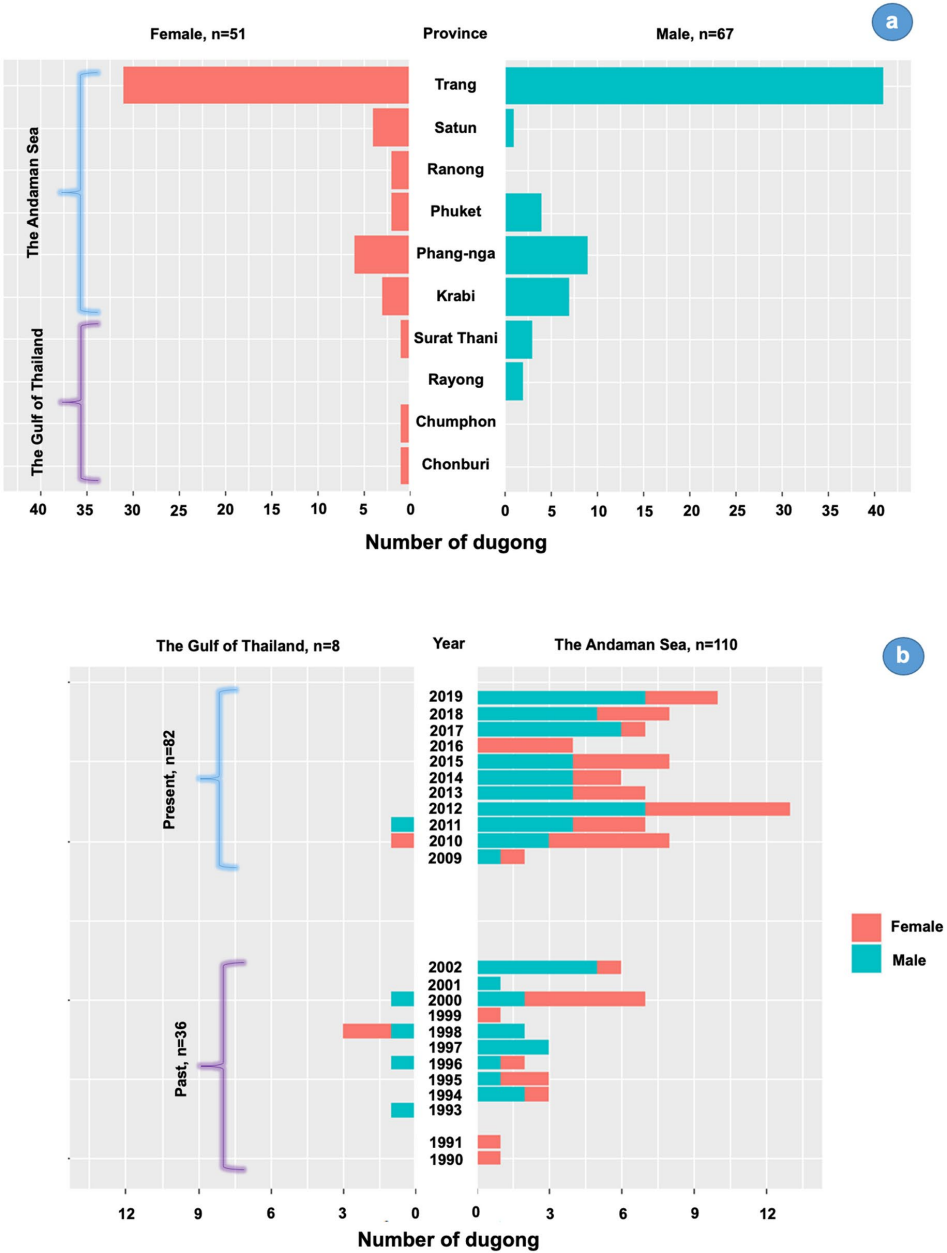
Number of samples from male and female dugong collected from 10 provinces in the Andaman Sea and Gulf of Thailand (**a**) and years of skin sample collection (**b**).

The Phuket Marine Biological Center, Phuket, Thailand provided skin samples from 118 deceased dugongs (male = 67, female = 51) that were stranded between 1990–2019 (Fig. 1, Supplementary Table S1). The samples were collected and preserved in 95% ethanol at − 20 °C. Additional preliminary data included stranding location, sex and body length. Use of banked samples meant animal ethics committee approvals were not needed.

### DNA extraction

Skin samples were extracted using DNA extraction kits according to manufacturer’s instructions (DNeasy Blood & Tissue Kit, Qiagen, Germany) at the Faculty of Veterinary Medicine, Chiang Mai University, and the DNA measured qualitatively and quantitatively by agarose gel electrophoresis and spectrophotometry, respectively^16^.

### Inter-simple sequence repeat (ISSR)

Thirty-four ISSR primers of the microsatellite UBC primer set obtained from the University of British Columbia, Vancouver, Canada were screened twice using a polymerase chain reaction (PCR) technique resulting in the selection of 13 primers that produced reproducible and unambiguous bands (Table 1). Five samples were amplified individually for screening by PCR and these consisted of 1× ViBuffer S (16 mM (NH_2_)^4^SO_4_, 50 mM Tris–HCl, 1.75 mM MgCl_2_, and 0.01% TritonTM X-100), 5 µm dNTP (Vivantis, Selangor Darul Ehsan, Malaysia), 0.2 µM ISSR primer, 1U Taq DNA polymerase (Vivantis, Selangor Darul Ehsan, Malaysia), and 20 ng DNA template with deionized water added to a volume of 25 µm. For every PCR reaction, deionized water was use instead of the DNA template to serve as a negative control. PCR amplifications were performed in PTC-200 at DNA EngineThermal Cycler (Bio-Rad Laboratories, Inc., CA, USA) under the following conditions: pre-denaturation at 95 °C for 5 min, followed by 37 cycles consisting of a denaturation step at 95 °C for 30 s, an annealing step at 58 °C for 45 s, and an extension step at 72 °C for 1 min with a final extension step at 72 °C for 10 min. The PCR products were stained by REDSAFE Nucleic acid staining solution (iNtRON Biotechnology, Gyeonggi-do, South Korea) and then separated electrophoretically on 2% agarose gel (PanReac AppliChem ITW companies, Darmstadt, Germany) by POWERPAC 200 (Bio-Rad, CA, USA) containing 1× Tris–acetate-ethylenediaminetetraacetate (TAE) buffer at 120 V for 30 min. The PCR products were then visualized by UV light under a GELMAX 125Imager (UVP, Cambridge, England).

**Table 1:** Nucleotide sequences of inter-simple sequence repeat primers obtained from University of British Columbia, Canada.

Primers	Sequence (5′–3′)	Length
UBC801	ATA TAT ATA TAT ATA TT	17-mer
UBC802	ATA TAT ATA TAT ATA TG	17-mer
UBC803	ATA TAT ATA TAT ATA TC	17-mer
UBC805	TAT ATA TAT ATA TAT AC	17-mer
UBC807	AGA GAG AGA GAG AGA GT	17-mer
UBC808	AGA GAG AGA GAG AGA GC	17-mer
UBC809	AGA GAG AGA GAG AGA GG	17-mer
UBC811	GAG AGA GAG AGA GAG AC	17-mer
UBC814	CTC CTC TCT CTC TCT A	16-mer
UBC817	CAC ACA CAC ACA CAC AA	17-mer
UBC818	CAC ACA CAC ACA CAC AG	17-mer
UBC822	TCT CTC TCT CTC TCT CA	17-mer
UBC823	TCT CTC TCT CTC TCT CC	17-mer
UBC824	TCT CTC TCT CTC TCT CG	17-mer
UBC825	ACA CAC ACA CAC ACA CT	17-mer
UBC826	ACA CAC ACA CAC ACA CC	17-mer
UBC827	ACA CAC ACA CAC ACA CG	17-mer
UBC835	AGA GAG AGA GAG AGA GYC	18-mer
UBC844	CTC TCT CTC TCT CTC TRC	18-mer
UBC845	TCT CTC TCT CTC TCT CRG	18-mer
UBC847	CAC ACA CAC ACA CAC ARC	18-mer
UBC848	CAC ACA CAC ACA CAC ARG	18-mer
UBC861	ACC ACC ACC ACC ACC ACC	18-mer
UBC866	CTC CTC CTC CTC CTC CTC	18-mer
UBC868	GAA GAA GAA GAA GAA GAA	18-mer
UBC869	GTT GTT GTT GTT GTT GTT	18-mer
UBC872	GATA GATA GATA GATA	16-mer
UBC874	CCCT CCCT CCCT CCCT	16-mer
UBC876	GATA GATA GACA GACA	16-mer
UBC880	GGA GAG GAG AGG AGA	15-mer
UBC881	GGG TGG GGT GGG GTG	15-mer
UBC892	TAG ATC TGA TAT CTG AAT TCC C	22-mer
UBC899	CAT GGT GTT GGT CAT TGT TCC A	22-mer
UBC900	ACT TCC CCA CAG GTT AAC ACA	21-mer

### Control region (D-loop) primer and amplification

One pair of PCR primers, DugDLF (5′-CAT ATT ACA ACG GTC TTG TAA ACC-′’) and DugDLR (5′-GTC ATA AGT CCA TCG AGA TGT C-3′) that contained 615 bp long regions of mtDNA d-loop (dugong) were used for amplification^17^. PCR reactions were conducted in 25 μl reaction volumes using Platinum Taq DNA polymerase (Invitrogen) and 10 × reaction buffer. Reactions generally contained 2 mM MgCl_2_, 0.4 mg/ml bovine serum albumin, 0.25 mM dNTPs, 0.4 μM forward and reverse primers, and 2 μl of DNA template. The PCR conditions were as follows: 95 °C for 5 min, 40 cycles of (95 °C for 30 s, 50 °C for 45 s, 72 °C for 1 min), and 72 °C for 10 min. Sanger direct sequencing was performed by Ward Medic Ltd. Bangkok, Thailand. Sequences were edited and aligned manually using the program MEGA-X version 10.1.8^18^ (GenBank accession numbers: MT542517-MT542637; Steller’s sea cow *Hydrodamalis gigas*, MH 717817).

### Statistical analysis

#### Data analysis of ISSR

Only clearly observed and unambiguous fragments were scored in a binary manner for band presence (1) or absence (0), and a binary matrix was generated to determine the level of polymorphism for each primer represented by the percentage of polymorphic bands^19,20^.

For ISSR, comparisons were made between two collection time periods: past (samples collected from 2009 to 2019; 10 years, n = 82) and present (samples collected from 1990 to 2002; 12 years, n = 36)^21,22^; and five location zones (Supplementary Fig. S1): upper Gulf of Thailand (Zone 1), lower Gulf of Thailand (Zone 2), upper Andaman Sea (Zone 3), middle Andaman Sea (Zone 4), and lower Andaman Sea (Zone 5). Observed number of alleles (Na), effective number of alleles (Ne), Shannon’s information index (I), observed heterozygosity (Ho), and expected heterozygosity (He) were analyzed by GENALEX program version 6.5^23^.

#### Analysis of the mitochondrial D-loop

Thai dugong alignment consisted of 118 mtDNA control region sequences (454 bp) obtained from samples collected from the Andaman Sea (n = 110) and Gulf of Thailand (n = 8). The global dugong alignment of 353 sequences (207 bp) included these Thai sequences and additional ones downloaded from GenBank (n = 235)^3,14,15,17,24,25^. The sequences were arranged into global dugong distributions representing the Pacific, Southeast Asia, South Asia and Southwest Indian Ocean regions (Supplementary Table S2).

The genetic structures within Thai dugong populations and genetic clades of global dugong populations were determined using three approaches:

(i) Genetic structure was estimated using MRBAYES program version 3.2.7a^26^ to identify the population clusters within both the global and Thai dugong alignment datasets.
(ii) Median joining haplotype network was constructed using program POPART version 1.7^27^ to assess the genetic lineage within the global dugong alignment as well as for the Thai dugong dataset. Haplotype network calculations were carried out for both analyses by assigning equal weights to all the variable sites.
(iii) Phylogenetic relationships were assessed for both Thai and global dugong alignments. The best fit nucleotide substitution and partition schemes for the DNA dataset were selected using Bayesian Information Criterion; BIC for Thai dugong population^28^ implemented in program JMODELTEST^29^. The best-fit substitution model was found to be HKY + I. Phylogenetic analysis implemented in MRBAYES program version 3.2.7a^26^ was conducted using a Bayesian inference approach^30^. The chain length consisted of 2 million generations of Markov Chain Monte Carlo (MCMC) simulations with average standard deviation of split frequencies at 0.007437 (< 0.01), sampled every 5000 generations, with the first 50,000 runs discarded as burn-ins. Steller’s sea cow was kept as an outgroup in the phylogenetic analysis. Finally, INTERACTIVE TREE OF LIFE (ITOL) online program (https://itol.embl.de) was used to view and annotate the consensus phylogenetic tree. A posterior probability value ≥ 0.05 was considered a strong relationship^31^.

For the global dugongs, the procedure was the same. The best fit nucleotide substitution and partition schemes for the DNA D-loop dataset of global dugongs were selected using the Akaike Information Criterion: AIC^32^ implemented in program JMODELTEST^29^. The best-fit substitution model was found to be TrN + G. Phylogenetic analysis implemented in program MRBAYES version 3.2.7a^26^ was conducted using a Baysian inference approach. The chain length consisted of 21 million generations of MCMC (Markov Chain Monte Carlo) simulation with an average standard deviation of split frequencies at 0.009924 (< 0.01), sampled every 5000 generations, with the first 50,000 runs discarded as burn-ins. Steller’s sea cow was kept as an outgroup in the phylogenetic analysis.

#### Bayesian skyline analyses and clade divergence dating

To reconstruct the demographic history of Thai dugongs, we ran a coalescent Bayesian skyline analysis in BEAST version 2.2.0^33^, where the changes in effective population size (Ne) over time were tested. This enabled past demographic changes of dugongs in Thailand to be inferred from the current patterns of genetic diversity within a population^34^. The number of samples from the Gulf of Thailand was small (n = 8), so only samples from the Andaman Sea (n = 110) were analyzed. Those sequences were used for analyses at a mutation rate of 2 × 10^–8^ bp^−1^ year^−1^^35^. The input was prepared in BEAUti. The analysis was run for 10^8^ iterations with a burn-in of 10^7^ with sampling every 10^4^ and a strict molecular clock. All operators were automatically optimized and the results were generated using TRACER version 1.7.1^36^. A maximum clade credibility tree was constructed from the resulting Bayesian trees using TREEANNOTATOR version 2.4.4 and FIGTREE version 1.4.3^3^. The same analysis parameters were used in an Extended Bayesian Skyline Plot analyses^37^ to estimate changes in population size over time for each of the major mtDNA clades. A 95% HPD (highest posterior density) distribution of the inferred number of population changes was used. Six mammalian species were used to compare data results, including rock hyrax (*Procavia capensis*), African elephant (*Loxodonta africana*), Asian elephant (*Elephas maximus*), West Indian manatee (*Trichechus manatus*), African manatee (*Trichechus senegalensis*), and Steller’s sea cow.

## Results

### ISSR

#### Polymorphism

Based on 13 ISSR primers, dugongs from the Andaman Sea and the Gulf of Thailand had similar percent polymorphic bands at 63.79% and 62.43%, respectively (Table 2). Primer UBC811 generated the highest value for percent polymorphic bands at 85.71% from both habitats. By contrast, primer UBC825 generated the lowest value of percent polymorphic bands at 33.33%.

**Table 2:** The percentage of polymorphic bands using different inter-simple sequence repeat primers.

Markers	Primer sequence	Male		Female		AND	GOT
		AND	GOT	AND	GOT		
UBC807	(AG)_8T_	75.00	75.00	50.00	33.33	75.00	75.00
UBC808	(AG)_8C_	54.55	54.55	72.73	55.56	72.72	72.72
UBC811	(GA)_8C_	57.14	57.14	85.71	57.14	85.71	85.71
UBC817	(CA)_8A_	50.00	44.44	70.00	50.00	70.00	60.00
UBC818	(CA)_8G_	50.00	50.00	75.00	66.67	75.00	75.00
UBC825	(AC)_8T_	33.33	33.33	33.33	33.33	33.33	33.33
UBC826	(AC)_8C_	30.00	30.00	50.00	50.00	50.00	50.00
UBC827	(AC)_8G_	50.00	50.00	60.00	60.00	66.67	66.67
UBC848	(CA)_8RG_	37.50	37.50	57.14	57.14	62.50	62.50
UBC866	(CTC)_6_	50.00	50.00	61.54	46.15	61.54	53.85
UBC874	(CCCT)_4_	33.33	33.33	55.56	42.86	55.56	55.56
UBC880	(GGAGA)_3_	50.00	50.00	66.67	66.67	66.67	66.67
UBC881	(GGGTG)_4_	45.45	40.00	54.55	45.45	54.55	54.55
Mean of percentage of polymorphic bands		47.41	46.56	60.94	51.10	63.79	62.43

*AND* Andaman Sea, *GOT* Gulf of Thailand.

Dugongs from the Andaman Sea had a higher percent polymorphic band value than those in the Gulf of Thailand for both sexes. Males had values of 47.41% and 46.56% from Andaman Sea and Gulf of Thailand, while females had values of 60.94% and 51.10%, respectively. DNA fragments were most pronounced in male dugongs in both habitats, of which 75% were polymorphic using UBC807. Meanwhile, in females, UBC811 produced 85.71% polymorphic bands from the Andaman Sea and 66.67% using UBC818 and UBC880 from the Gulf of Thailand.

### Genetic diversity

#### Temporal genetic diversity

All values (Na, N_e_, *I*, H_o_, H_e_ and F_st_) indicated higher genetic variation in the present (2009–2019) compared to the past (1990–2002) periods for dugongs in both the Andaman Sea and Gulf of Thailand (Table 3).

**Table 3:** Diversity indices values (mean ± SE) between past (1990–2002) and present (2009–2019) time periods for dugongs in the Andaman Sea and Gulf of Thailand.

Location	Period	N_a_	N_e_	*I*	H_o_	H_e_	F_st_
Andaman Sea	Past (n = 30)	17.692 ± 0.593	12.119 ± 0.323	2.654 ± 0.028	0.997 ± 0.003	0.917 ± 0.002	− 0.088 ± 0.003
	Present (n = 80)	17.077 ± 0.400	11.272 ± 0.329	2.571 ± 0.026	0.972 ± 0.007	0.910 ± 0.003	− 0.068 ± 0.007
The Gulf of Thailand	Past (n = 6)	8.923 ± 0.329	7.682 ± 0.372	2.108 ± 0.044	0.974 ± 0.026	0.866 ± 0.007	− 0.124 ± 0.026
	Present (n = 2)	3.769 ± 0.122	3.692 ± 0.162	1.306 ± 0.042	0.885 ± 0.061	0.721 ± 0.015	− 0.210 ± 0.065

*N*_*a*_ observed number of alleles, *N*_*e*_ effective number of alleles, *I* Shannon’s information index, *H*_*o*_ observed heterozygosity, *H*_*e*_ expected heterozygosity.

#### Spatial genetic diversity

Genetic variation of dugongs in Zone 1 (Na, N_e_, *I*, H_o_, H_e_ and F_st_) was the lowest compared to all other zones, with that in Zone 5 being the highest (Table 4).

**Table 4:** Diversity indices values (mean ± SE) for dugongs in the five regional zones.

Zone	N_a_	N_e_	*I*	H_o_	H_e_	F_st_
1 (n = 3)	4.923 ± 0.329	4.795 ± 0.357	1.547 ± 0.078	1.000 ± 0.000	0.774 ± 0.021	− 0.305 ± 0.039
2 (n = 5)	7.615 ± 0.140	6.508 ± 0.251	1.949 ± 0.029	0.923 ± 0.036	0.843 ± 0.007	− 0.094 ± 0.040
3 (n = 11)	10.308 ± 0.524	8.250 ± 0.416	2.197 ± 0.053	0.984 ± 0.011	0.875 ± 0.007	− 0.126 ± 0.015
4 (n = 20)	13.462 ± 0.447	8.631 ± 0.266	2.350 ± 0.033	0.983 ± 0.007	0.883 ± 0.003	− 0.114 ± 0.009
5 (n = 79)	17.846 ± 0.576	11.688 ± 0.394	2.608 ± 0.034	0.976 ± 0.003	0.913 ± 0.003	− 0.069 ± 0.005

*N*_*a*_ observed number of alleles, *N*_*e*_ effective number of alleles, *I* Shannon’s information index, *H*_*o*_ observed heterozygosity, *H*_*e*_ expected heterozygosity.

### Genetic differentiation

The pairwise Nei’s genetic distance analysis showed the lowest distance was between past and present periods in the Andaman Sea (0.140) and the highest was between past and present periods in the Gulf of Thailand (0.940) (Table 5). The pairwise Nei’s genetic identity analysis showed the closest identity was between past and present periods in Andaman Sea (0.870) and the farthest identity was between past and present periods in the Gulf of Thailand (0.063) (Table 5).

**Table 5:** Pairwise population matrix of Nei’s genetic distance and identity in past (1990–2002) and present (2009–2019) time periods for dugongs in the Andaman Sea and Gulf of Thailand.

	Andaman Sea		The Gulf of Thailand	
	Past	Present	Past	Present
**Andaman Sea**				
Past				
Present	0.140/0.870			
**The Gulf of Thailand**				
Past	0.406/0.666	0.410/0.664		
Present	0.542/0.458	0.499/0.507	0.940/0.063	

Data present as distance/identity.

The pairwise Nei’s genetic distance analysis showed the lowest distance between Zones 4 and 5 in the Andaman Sea (0.146) and the highest between Zones 1 and 2 in the Gulf of Thailand (1.151) (Table 5). The pairwise Nei’s genetic identity analysis showed the closest identity was between Zones 4 and 5 in the Andaman Sea (0.864) and the farthest identity was between Zones 1 and 2 in the Gulf of Thailand (0.316) (Table 6).

**Table 6:** Pairwise population matrix of Nei’s genetic distance and identity for dugongs in the five regional zones.

	Zone				
	1	2	3	4	5
**Zone**					
1					
2	1.151/0.316				
3	0.818/0.441	0.530/0.588			
4	0.615/0.540	0.425/0.654	0.419/0.658		
5	0.605/0.546	0.346/0.708	0.210/0.810	0.146/0.864	

Data present as distance/identity.

### Mitochondrial D-loop of Thai dugongs

#### Phylogenetic tree for Thai dugongs

**Figure 2:**
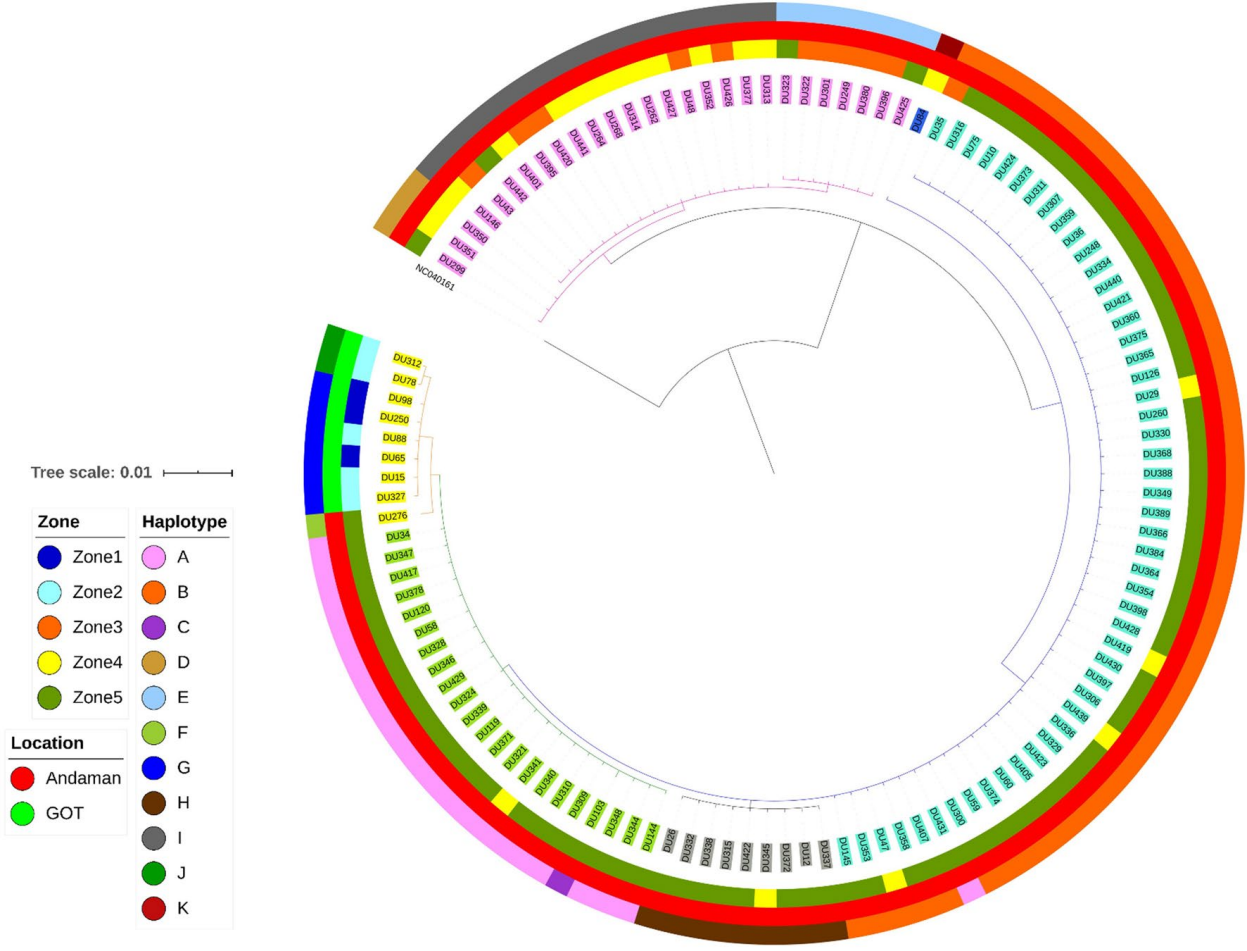
Phylogenetic tree of dugongs in the Andaman Sea and Gulf of Thailand. Two main clades were identified: 27 dugongs were grouped in clade A and 91 were grouped in clade B.

The phylogenetic tree of dugongs in this study from mitochondrial D-loop typing (Fig. 2) showed two main clades: clade A (n = 27) and clade B (n = 91).

**Figure 3:**
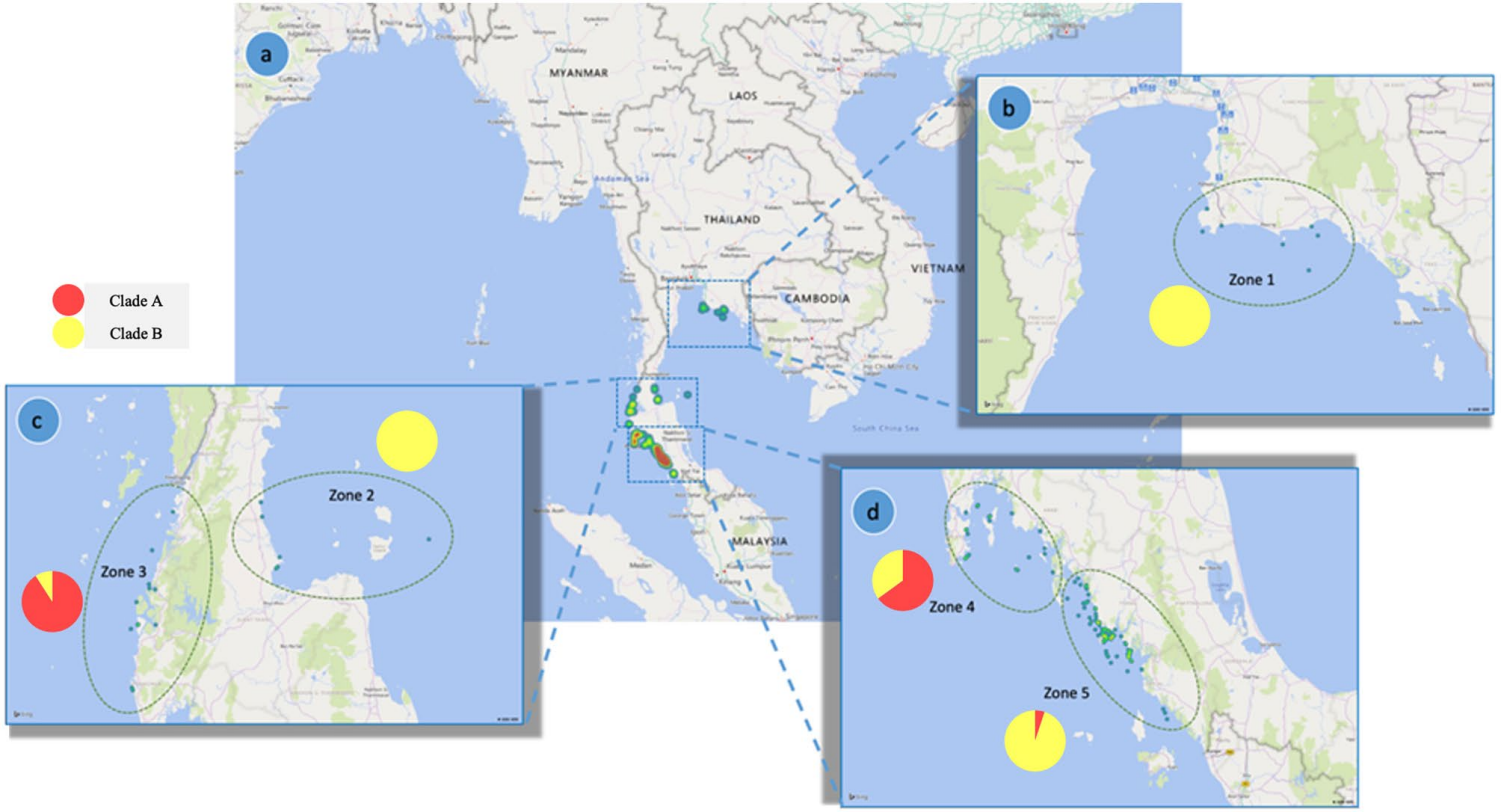
Geographic distribution of dugongs separated by clades in the study area. Zone 1, upper the Gulf of Thailand (**a**,**b**); Zone 2, lower Gulf of Thailand (**a**,**c**); Zone 3, upper Andaman Sea (**a**,**c**), Zone 4; middle Andaman Sea (**a**,**d**); and Zone 5; lower Andaman Sea (**a**,**d**). Background images were generated using the ‘Map’ tool in Microsoft Excel.

Phylogeographics by clade (Fig. 2) showed that clade A included haplotypes D, E and I and was dispersed across three zones in the Andaman Sea, while clade B consisted of haplotypes A, B, C, F, G, H, J and K and was found widely distributed in both the Andaman Sea and Gulf of Thailand (Fig. 3). When comparing the proportions between clades in Andaman Sea, clade A had a larger proportion in Zones 3, 4 and 5. In the Gulf of Thailand, only clade B was found in both Zones 1 and 2.

#### Haplotypes of Thai dugongs

**Figure 4:**
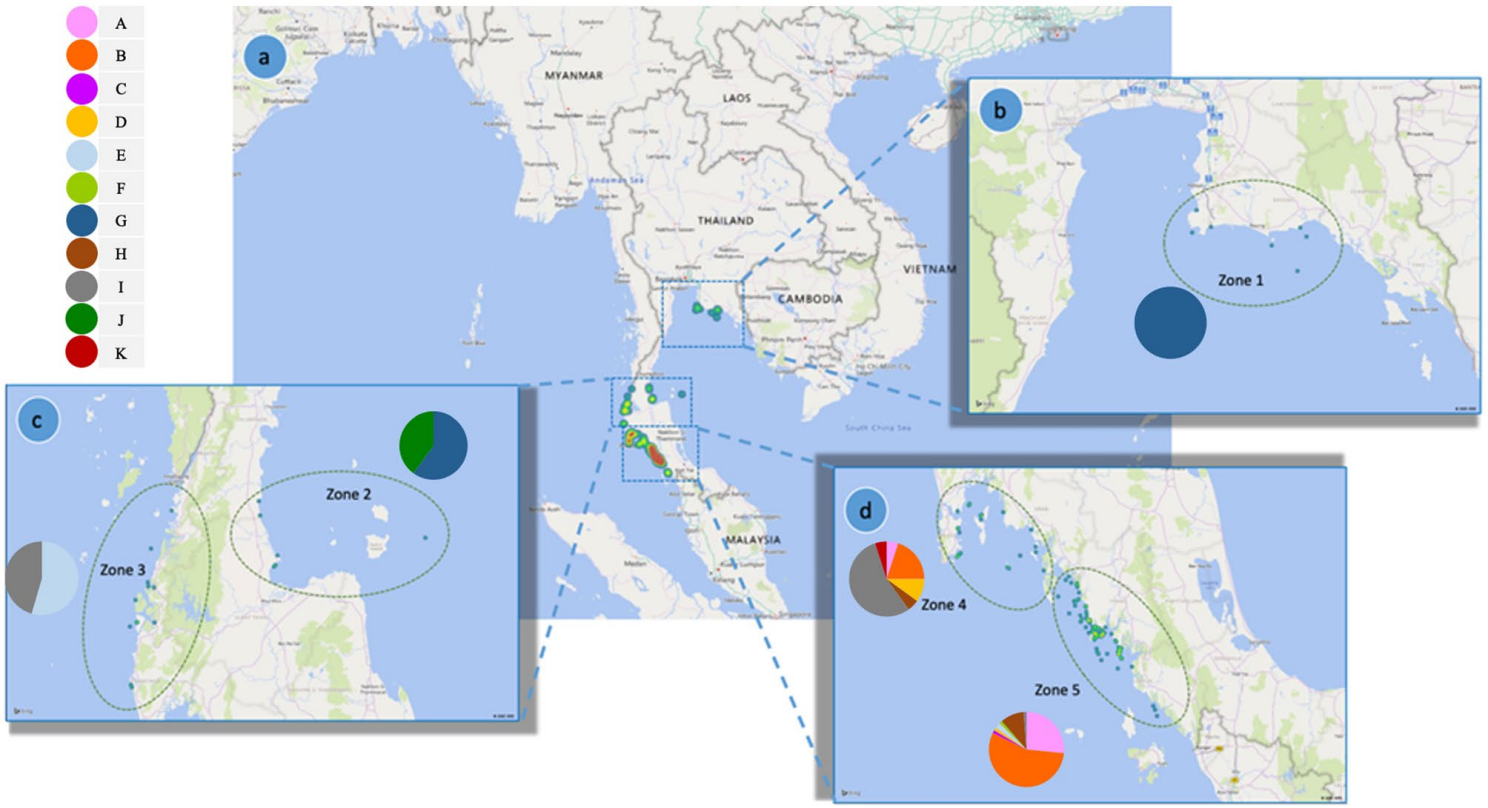
Distribution of haplotypes in the study area separated by Thailand sea zones. Zone 1 in the Gulf of Thailand had one haplotype (**a**,**b**), whereas Zones 2 and 3 each had two specific haplotypes (**a**,**c**). The regions with the highest haplotype patterns were Zones 4 and 5, with eight and six haplotypes, respectively (**a**,**d**).

Eleven median joining network haplotypes (A, B, C, D, E, F, G, H, I, J and K) were identified and shown in Supplementary Fig. S2. Phylogeographic by haplotype is shown in Fig. 4, and by clade in Fig. 2. The haplotypes of dugongs from the Andaman Sea and Gulf of Thailand were clearly distinguished from each other, with haplotypes G and J found only in the Gulf of Thailand. Haplotype J was uniquely found in Zone 2 (n = 2), while haplotype G was found in both Zones 1 (n = 3) and 2 (n = 3). In the Andaman Sea, haplotype C (n = 1) and F (n = 1) were found only in Zone 5, and haplotype K was only in Zone 4 (n = 1). Haplotypes A (n = 1, 21), B (n = 4, 44), D (n = 2, 1) and H (n = 1, 8) were found in Zones 4 and 5. Haplotype E was found in Zones 3 (n = 6) and 5 (n = 2), while haplotype I found in Zones 3 (n = 5), 4 (n = 11) and 5 (n = 1). Thus, only haplotype I was found throughout all zones in the Andaman Sea (Fig. 4).

### Bayesian skyline plots

**Figure 5:**
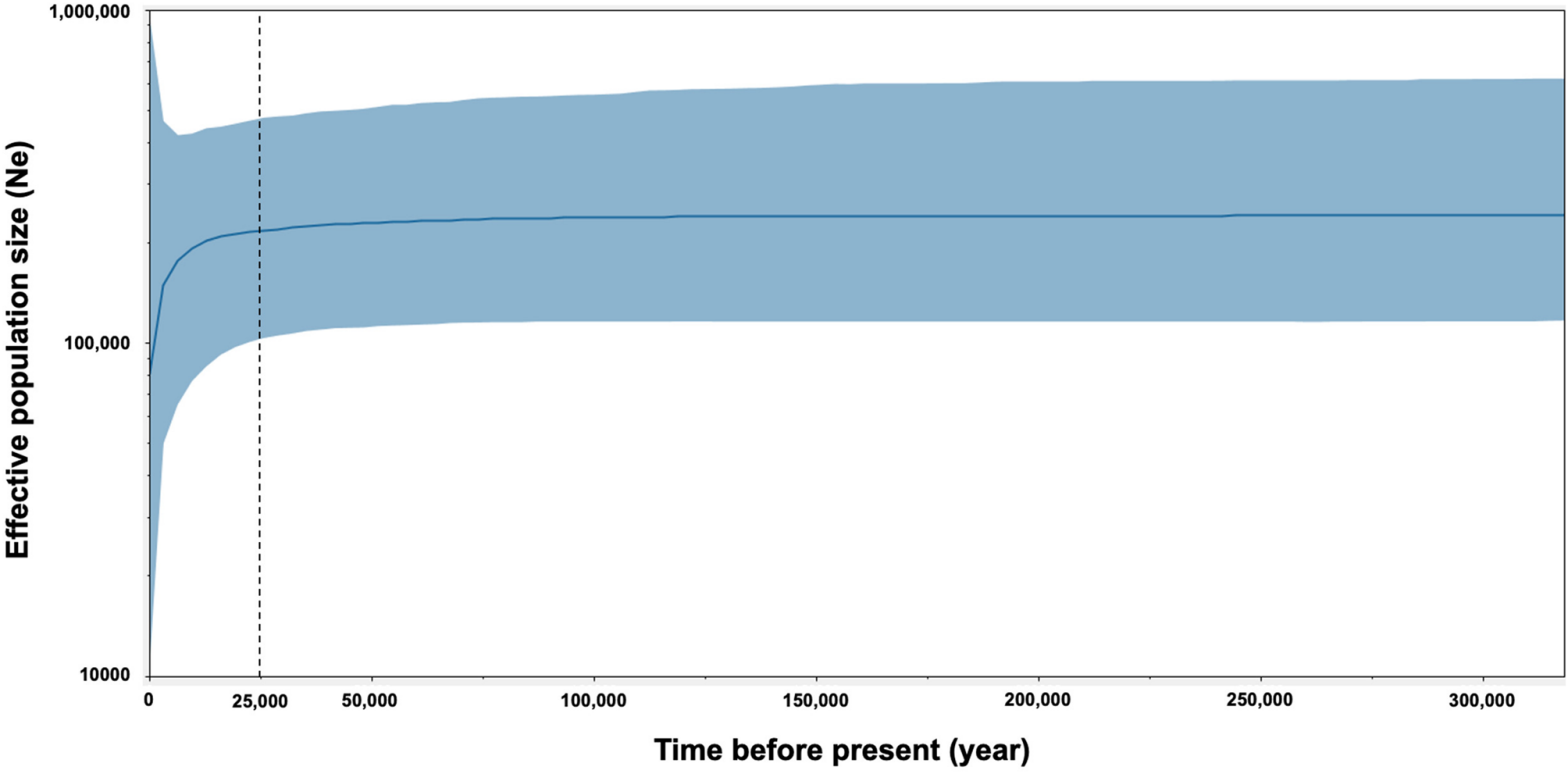
Bayesian Skyline Plots of the D-loop marker of Andaman zone. The Y-axis indicates effective population size, while the X-axis indicates mean time in millions of years before the present. The thick line represents the median, the blue band represent the standard error and black dashed line represent at 25,000 years ago when dugong population numbers began to decline.

Due to the low number of samples from the Gulf of Thailand, BSP could not be calculated. Moreover, all samples from the three zones in the Andaman Sea were merged for calculating BSP from D-loop markers (Fig. 5). The calculation of BSP was done using a mutation rate = 2% Myr^38^, which indicated that 2 bp from 100 bp mutate every 1 million years. Additionally, the result revealed that the effective population size of dugongs was stable from 300,000 to about 25,000 years ago, after which it has gradually decreased.

### Mitochondrial D-loop of global dugong

#### Global dugong phylogenetic tree

**Figure 6:**
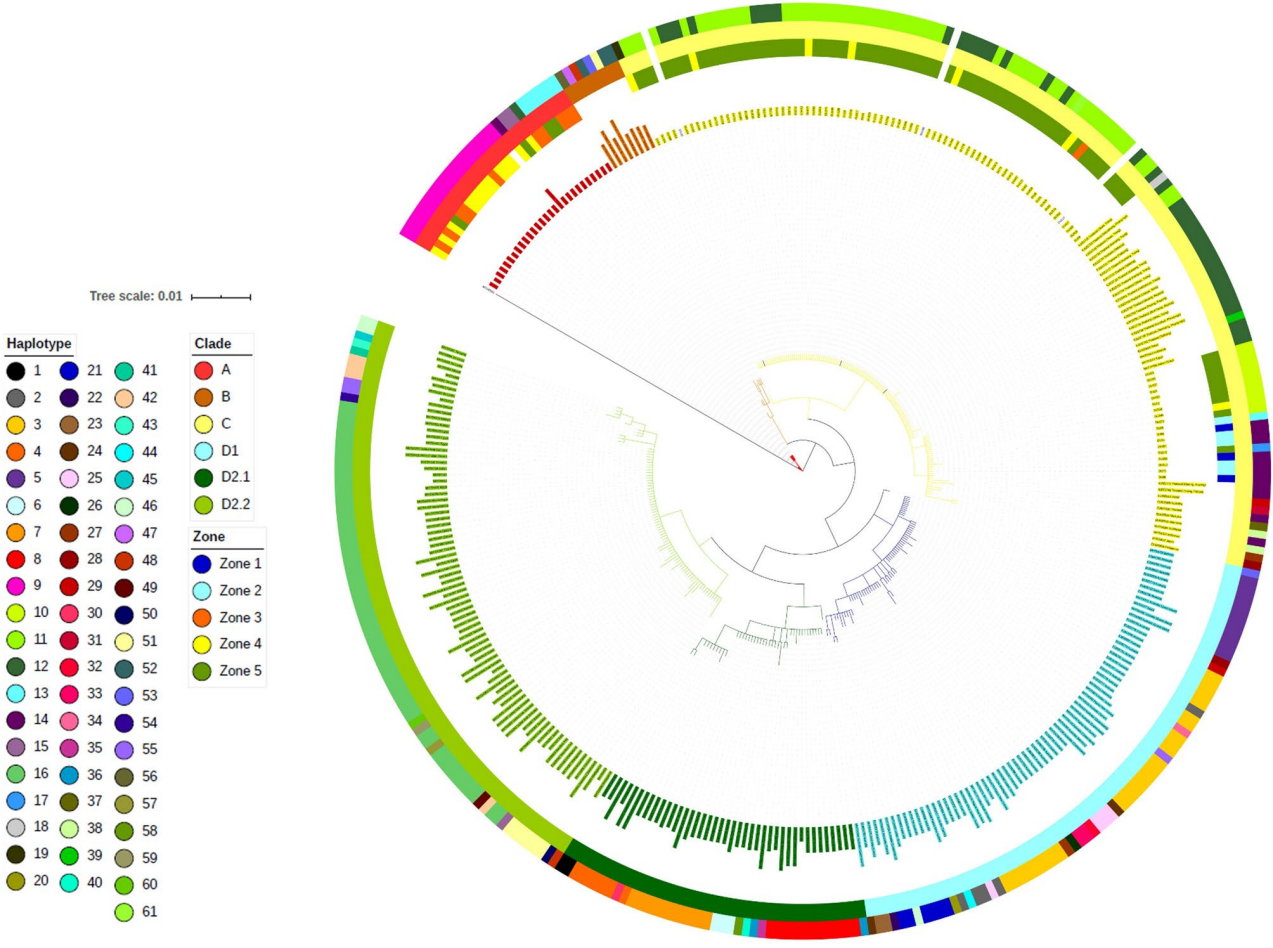
Phylogenetic tree of global dugong haplotypes, locations and zones. Dugong populations were divided into five clades: A (28 sequences); B (8 sequences); C (126 sequences); D1 (71 sequences); D2.1 (41 sequences); and D2.2 (79 sequences). Background images were generated using the ‘Map’ tool in Microsoft Excel.

A total of 353 sequences are shown in the phylogenetic dendrogram (Fig. 6). Five clades were categorized according to dugong population: A (28 sequences); B (8 sequences); C (126 sequences); D1 (71 sequences); D2.1 (41 sequences); and D2.2 (79 sequences). There were 28 dugong mtDNA d-loop sequences in clade A, which were restricted to Thailand and consisted of one sequence from the study of Bushell (2013), 27 sequences from this study from Andaman Sea, segregated into 10 sequences (37%), 13 sequences (48%) and 4 sequences (15%) from Zone 3, 4 and 5, respectively, all entirely found in Thailand.

### The world haplotype

**Figure 7:**
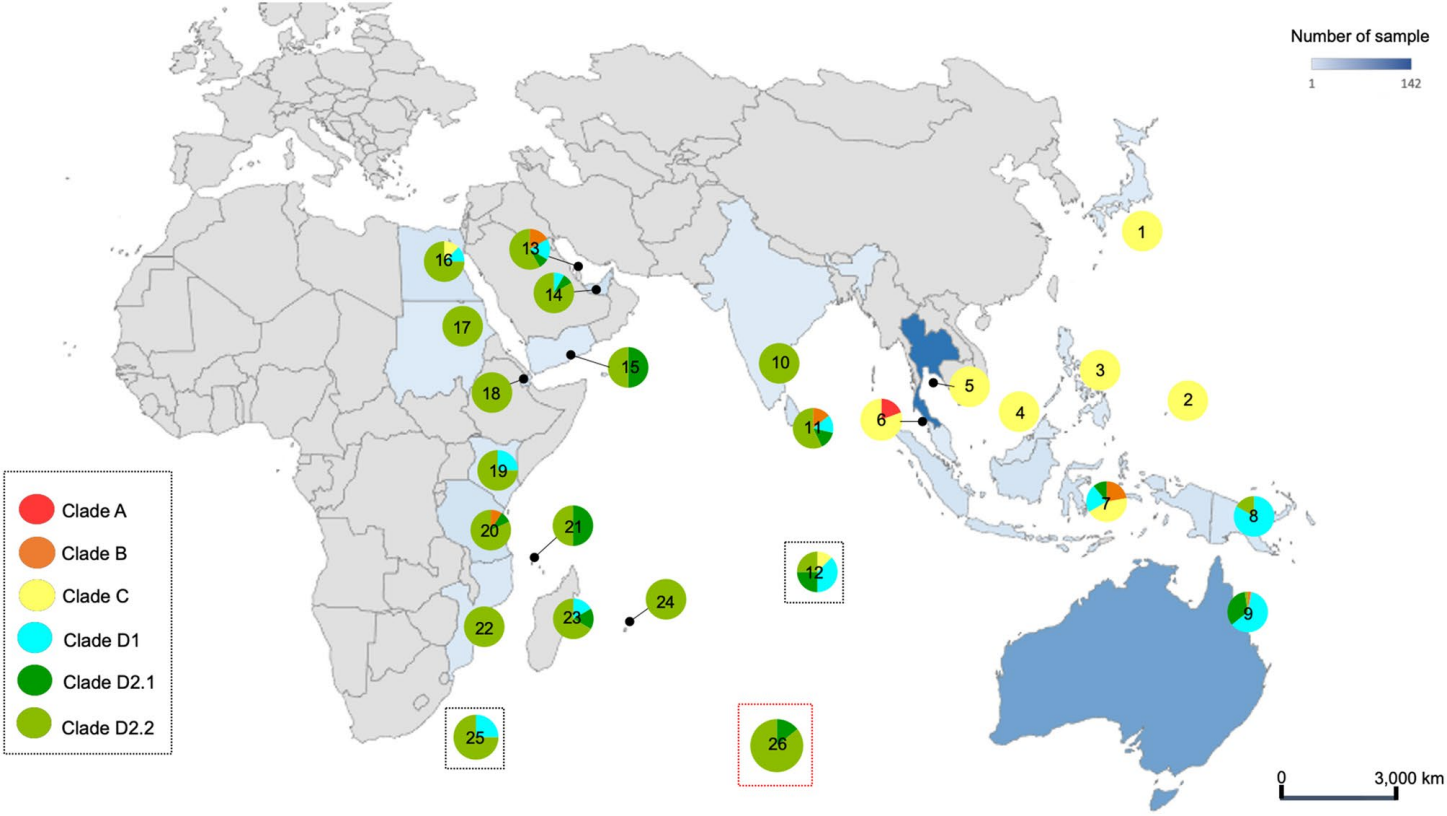
Geographic distribution global dugong clades across 26 locations. Japan (1 clade), Palau (2), Philippines (3), Malaysia (4), Thailand-Gulf of Thailand (5), Thailand-Andaman Sea (6), Indonesia (7), Papua New Guinea (8), Australia (9), India (10), Sri Lanka (11), Indian Ocean (12), Bahrain (13), United Arab Emirates (14), Yemen (15), Egypt (16), Sudan (17), Djibouti (18), Kenya (19), Tanzania (20), Comoros (21), Mozambique (22), Madagascar (23), Mauritius (24), Indian Ocean in Africa (25) and Unknown (26). Background images were generated using the ‘Map’ tool in Microsoft Excel.

A total of 61 haplotypes were generated from Median joining network (Figs. 7 and Supplementary Fig. S3). Hap 9, Hap 13 and Hap 15 were found in clade A. Clade B consisted of Hap 48, Hap 52, Hap 53 and Hap 57 were found in three continents: Asia (Bahrain, Indonesia and Sri Lanka), Oceania (Australia) and Africa (Tanzania). Clade C consisted of 13 haplotypes mostly (10 haplotypes) in Thailand, Indonesia, Japan, Malaysia and Philippines, with only a few (3 haplotypes) in the Indian Ocean, Oceania and Pacific Islands. Clade D1 included Australia, Papua New Guinea, some countries in Asia (Bahrain, Indonesia, Sri Lanka, United Arab Emirates), Indian Ocean and some countries in Africa (Egypt, Madagascar, Kenya) and consisted of 18 haplotypes (Hap 2, Hap 3, Hap 5, Hap 21, Hap 22, Hap 23, Hap 24, Hap 25, Hap 26, Hap 27, Hap 29, Hap 33, Hap 34, Hap 35, Hap 45, Hap 47, Hap 54 and Hap 56). One unknown sequence was discovered in clade D2.1 with Australia, Yemen and Comoros along with 10 haplotypes (Hap 1, Hap 4, Hap 6, Hap 7, Hap 8, Hap 31, Hap 36, Hap 37, Hap 41 and Hap 59). Moreover, six unknown sequences and another 13 haplotypes were reported in clade D2.2 that consisted of Australia, Papua New Guinea, Bahrain, Sri Lanka, United Arab Emirates, India, Indian Ocean, Egypt, Madagascar, Kenya, Africa, Comoros, Mozambique, Mauritius, Sudan, Tanzania, and Djibouti.

### World clade divergence dating

**Figure 8:**
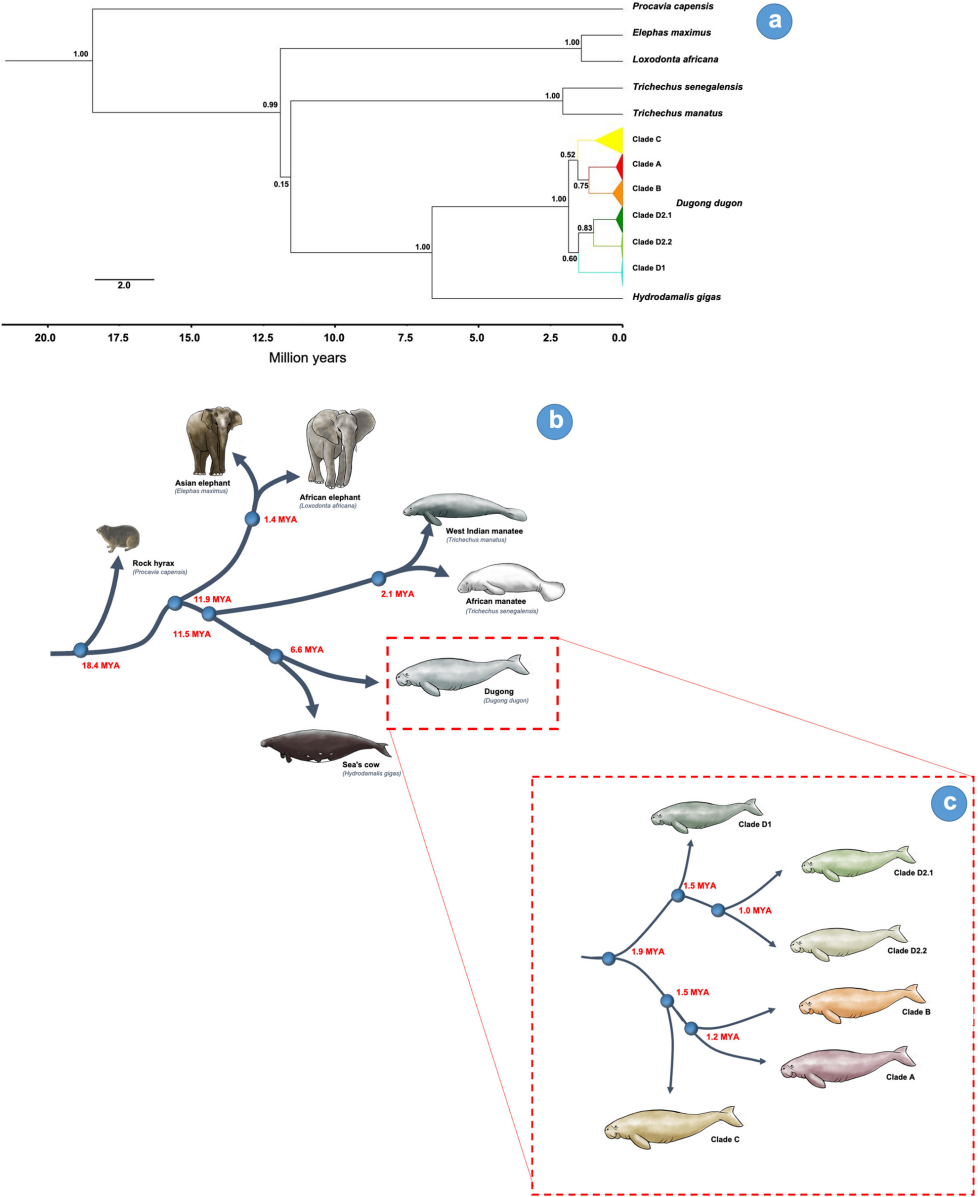
Maximum clade credibility tree for dugong mtDNA haplotypes showing estimated ages of clade MRCAs (most recent common ancestors). Compiled from Bayesian MCMC analyses implemented in BEAST. Bayesian clade posterior probabilities are indicated on nodes. Node ages are presented as median node heights with 95% HPD intervals represented by bars (**a**). The separated time (million years ago; MYA) of seven mammalian species for rock hyrax, African and Asian elephant, West Indian and African manatee, Steller’s sea cow and dugong. The separated time (million years ago; MYA) for each clade of dugongs (**c**). The animals were drawn by the author, Promporn Piboon.

The mtDNA control region sequences of dugongs in this study revealed that the population divided into two groups approximately 1.9 million years ago. The first group consisted of dugongs classified in clades D1, D2.1 and D2.2. Clade D1 separated from the group 1.5 million years ago, while clades D2.1 and D2.2 separated from the group about 1.0 million years ago. In the second group, dugongs were classified in clades A, B and C. Clade C emerged 1.5 million years ago followed about 0.3 million years later by separation into clades A and B (Fig. 8).

## Discussion

It is well-known that dugongs are threatened with extinction throughout their habitat ranges, due primarily to human disturbances. Creating insurance populations of dugongs in captivity has not been successful, so it is important to understand genetic structure and diversity of wild dugong populations to evaluate the status of this species and how it varies across regions and time. Results of this study support our hypothesis that genetic diversity of dugongs in the coastal areas of Thailand has decreased over time although we recognize this is based on relatively small sample sizes, so more work is needed. Moreover, from our phylogeographic analysis, 28 of 118 dugongs were in a clade that diverged over a million years ago and found only in Thailand, and as such may need special conservation protection.

### Genetic diversity over time

Aerial surveys are the most popular technique used to determine dugong numbers^21,39,40^. The last survey of the Andaman Sea and the Gulf of Thailand was in 2017 and identified 221 individuals^11^. However, these numbers were only approximates. Analysis of genetic diversity can be a more powerful tool to evaluate the genetic structure of dugong populations that does not rely on knowing exact numbers of animals in an area. Our data found that genetic variation was higher in the past (1990–2002) compared to present (2009–2019) time periods in both seas. Reductions in population size and absence of gene flow can lead to reductions in genetic diversity, reproductive fitness, and a limited ability to adapt to environmental change, thus increasing the risk of extinction^41^. Likewise, from a previous review by Willoughby et al.^42^, reductions in heterozygosity and allelic richness based on microsatellite marker analyses have been observed in threatened species, suggesting that inbreeding and genetic drift are both effective at removing genetic diversity in endangered populations. Therefore, the decrease in genetic diversity of dugongs in Thailand over the past decades may reflect some inbreeding in the population that could ultimately impact fitness and survival.

Degradation of seagrass habitat is a concern for the sustainability of dugong populations. Seagrass areas in Thailand cover 255 square kilometers, distributed along the Andaman Sea and the Gulf of Thailand in 13 provinces, with 13 species of seagrasses identified as important food sources for endangered marine mammals such as dugongs^8,43–45^. Much of the degradation of seagrass beds is human-caused by coastal construction, pollution and illegal fishing^46^, but they have also been affected by seasonal changes in monsoons in some areas^47^. The genetic similarity of dugongs in the Andaman Sea, which has a long coastline, may mean there is more gene flow than those in more restricted areas. There does not appear to be a high level of inbreeding based on a negative fixation (Fst) value indicating more heterozygosity in this population. While the mating system of dugongs is poorly understood, it has been suggested that decreasing numbers may be impacting mating choices, leading to possible inbreeding in the future^48^.

### Genetic diversity between coastal regions

Dugongs living in the Andaman Sea had higher genetic diversity than those in the Gulf of Thailand. The population in Zone 5 (Trang and Satun area) had the highest genetic diversity, which might be because these two habitats have a larger number of dugongs in the population^11^. It is estimated that there are fewer dugongs living in the Gulf of Thailand (estimated number = 30) than in the Andaman Sea (estimated number = 191) in 2017^49^. Furthermore, records of 282 strandings from 1962 to February 2008 found 71.6% were from the Andaman Sea, 25.8% were from the Gulf; 2.6% had no information on the stranding place^13^. Genetic diversity reflects the total number of genetic characteristics and is related to the number of genes and their alleles within individuals and can influence adaptability and distribution of a species in diverse habitats. Nevertheless, the results of this study found good genetic variability within and between Thai waters. The results showed that the expected and observed heterozygosity (He and Ho) were high, which might suggest dugongs were moving between habitats^50^. The percentage of polymorphic bands indicated a high level of variability in female dugongs at 60.94% and 51.10% from the Andaman Sea and Gulf of Thailand, respectively, with lower values of 47.41% and 46.56% for males, similar to a previous genetic study using microsatellite markers^15^. The Shannon’s information index values (I) were higher in dugongs in the Andaman Sea (past = 2.654 ± 0.028, present = 2.562 ± 0.028) compared to those in the Gulf of Thailand (past = 2.108 ± 0.044, present = 1.783 ± 0.082). In addition, the genetic variability of dugongs from samples collected between 1990 and 2019 was higher than that of other species, such as Asian elephant (2.45 ± 0.05)^51^, domestic dog (2.59 ± 0.034)^20^, Holstein cattle (0.23 ± 0.23)^52^, sheep (0.31 ± 0.30)^52^, buffaloes (0.28 ± 0.11)^53^, and goat (0.39 ± 0.30)^54^ that ranged from 0.18 to 2.59^20,51–54^. The highest I index was observed in Zone 5 (2.608), which represents the highest dugong population. Dugongs in Zone 1 (1.547) in the Gulf of Thailand had the lowest I index, although dugong habitat and traveling distance are considered important factors for genetic variability as they inhabit a broad but fragmented range^50,55^. Although they are not considered migratory, they are known to travel great distances from one coastal area to another, and evidence from a previous study (Bushell, 2013) showed there was some migration around the Malaysian peninsula from the Andaman Sea to the Gulf of Thailand^15^. Thus, there may be a chance that dugongs from both seas can intermingle across zones, which would increase the genetic diversity of Thailand’s dugong populations. Moreover, the dugong’s generation time is around 27 years and violates assumptions of non-overlapping generations due to life span; that and their random mating behavior could also aid in maintaining high genetic variability and decrease the potential for inbreeding in small populations like the dugong^35,56^.

Fixation index (Fst) values in this study also were negative, which might indicate little genetic subdivision between populations; that is, there is only one species present in this population. Evolutionary forces can influence genetic differentiation—the accumulation of differences in allelic frequencies between completely or partially isolated populations, and so it is important to understand selection or genetic drift in endangered species^57^. Nei’s genetic distance was lowest in the Andaman Sea from past to present, and between zones potentially because of a limited ability to find mates when populations are small and spread out. In the future, dugongs in the Gulf of Thailand might have an inbreeding problem more than other populations because of low population numbers^15^.

The finding of some regional genetic differences agrees with data on other biological parameters. In 2017, Nganvongpanit and colleagues found skull morphometric analyses were 100% accurate in identifying dugongs in the Andaman Sea versus the Gulf of Thailand, and that dugongs living in the Andaman Sea were larger, based on skull size, than those in the Gulf of Thailand^58^. In addition, mineral elements in dugong teeth were significantly different between dugongs in the two habitats^59^. Thus our data supports the idea of regional biological distinctions, with haplotype differences between dugongs in the Andaman Sea (Hap A, B, C, D, E, F, H, I and K) and Gulf of Thailand (Hap G and J).

### Thai dugongs compared to global populations

Of great interest was the finding that the clade A group had characteristics found only in the Andaman Sea and restricted to Thailand, with decreasing numbers found among Zones 3, 4 and 5. Dugongs diverged 1.9 million years ago, similar to a previous study that also found the separation started about 2.0 million years ago^3^. By comparison, the Thai population separated from other groups around 1.2 million years ago. The reason for the separation might be because of geographical factors. The dugong is a marine mammal that lives in shallow waters, near the coast, and relies on seagrass for food^35,48^. It also has to come up to the surface to breathe every 1–2 min^35^. Dugong can travel as much as 100 km/day or more^8^, but movement is limited and restricted to areas with seagrass. For example, in adjacent waters, dugongs are found in the Bay of Bengal, but not the Gulf of Mataban, the latter of which has no seagrass distribution^60^. On the southern side to Malaysia is the Strait of Malacca where dugongs have not been reported. Although there is still some distribution of seagrass, it is considerably less than on the eastern side of Malaysia^60,61^. The Strait of Malacca is an important passageway between China and India and used heavily for commercial trade. It is narrow, contains thousands of islets, and is an outlet for many rivers. This region has been mentioned in ancient Chinese texts, as far back as the fourteenth century^62^. So, the dugong population has been restricted to the Andaman Sea of Thailand for a long time, with a limited ability to migrate. Therefore, it is a population group that has evolved with specific genetic characteristics.

A previous study reported that Southeast Asia and Indian Ocean regions each consisted of three dugong lineages^3^. But in this study, we found two clades in Southeast Asia and five clades in the western Indian Ocean, which might be due to differences in the number of sequences used and the length of those sequences. The previous study^3^ reported more variation among dugongs in the Southeast Asia region due to the longer sequence length used in the statistical analysis (355 bp), while our study used shorter sequences (207 bp). In another study, it was shown that longer sequences of D-loop mtDNA resulted in a higher genetic pattern^63^. Our study reported the highest genetic variation and the highest number of haplotypes were found in dugongs living in the Indian Ocean. This might because we had a higher number of sequences used for the calculation.

## Conclusion

This study provides the most thorough description of genetic groups of the dugong at a global scale to date and an in-depth investigation of genetic diversity and structure of Thai dugong populations, and shows the distribution of clades based on world geography. In general, genetic diversity in this study (using ISSR markers) was higher compared to studies using other dominant markers such as Random Amplified Polymorphic DNA (RAPD) marker or Amplified Fragment Length Polymorphism (AFLP) maker^15,64,65^.

This study filled in missing information on phylogeography of dugongs in the Asian region, including Thailand, Philippine, Palau and Japan. There also were new discoveries, including that only two genetic populations of dugongs exist in Thailand. Additionally, one of them has a specific haplotype restricted to Thailand. Because captive breeding has not been successful for increasing dugong populations, additional guidelines and laws are needed to conserve these rare populations of dugongs and stem the continued declines in both the Andaman Sea and Gulf of Thailand.

## Supplementary Information

Supplementary Figure S1.

Supplementary Figure S2.

Supplementary Figure S3.

Supplementary Table S1.

Supplementary Table S2.
