# Supplementary material for: Genetic diversity in a unique population of dugong (Dugong dugon) along the sea coasts of Thailand
Source: Sci Rep. 2021 Jun 2;11:11624. doi: 10.1038/s41598-021-90947-4 (PMC8172547; doi:10.1038/s41598-021-90947-4)
Supplement: Supplementary file 2 — Supplementary Figure S2. [file 41598_2021_90947_MOESM2_ESM.docx]

**Genetic diversity in a unique population of dugong (*Dugong dugon*) along the sea coasts of Thailand**

Anocha Poommouang^1^, Wannapimol Kriangwanich^1^, Kittisak Buddhachat^2,3^, Janine L. Brown^4^, Promporn Piboon^1^, Siriwadee Chomdej^5^, Jatupol Kampuansai^5^, Supamit Mekchay^6^, Patcharaporn Kaewmong^7^, Kongkiat Kittiwattanawong^7^, and Korakot Nganvongpanit ^1,2*^

^1^ Department of Veterinary Biosciences and Public Health, Faculty of Veterinary Medicine, Chiang Mai University, Chiang Mai 50100, Thailand.

^2^ Excellence Center in Veterinary Bioscience, Chiang Mai 50100, Thailand.

^3^ Department of Biology, Faculty of Science, Naresuan University, Phitsanulok 65000, Thailand.

^4^ Smithsonian Conservation Biology Institute, Center for Species Survival, 1500 Remount Road, Front Royal, VA 22630, USA.

^5^ Department of Biology, Faculty of Science, Chiang Mai University, Chiang Mai 50200 Thailand

^6^ Department of Animal and Aquatic Sciences, Faculty of Agriculture, Chiang Mai University, Chiang Mai 50200, Thailand

^7^ Phuket Marine Biological Center, Phuket 83000, Thailand.

Corresponding author

Prof. Dr. Korakot Nganvongpanit

Animal Bone and Joint Research Laboratory, Department of Veterinary Biosciences and Public Health, Faculty of Veterinary Medicine, Chiang Mai University, Chiang Mai 50100, Thailand

E-mail: korakot.n@cmu.ac.th Tel.: +66-53-948046

E-mail:

AP=anopotter@hotmail.com

WK=wannapimol.k@gmail.com

KB = k_buddhachat@yahoo.com

JB= BrownJan@si.edu

PP= muy_v3@hotmail.com

SC= siriwadee@yahoo.com

JK= Jatupol_k@hotmail.com

SM= supamit.m@cmu.ac.th

PK = marineanimal.vet@gmail.com

KK = kkongkiat@gmail.com

KN = korakot.n@cmu.ac.th

**Short title:** Genetic diversity in Thailand dugong


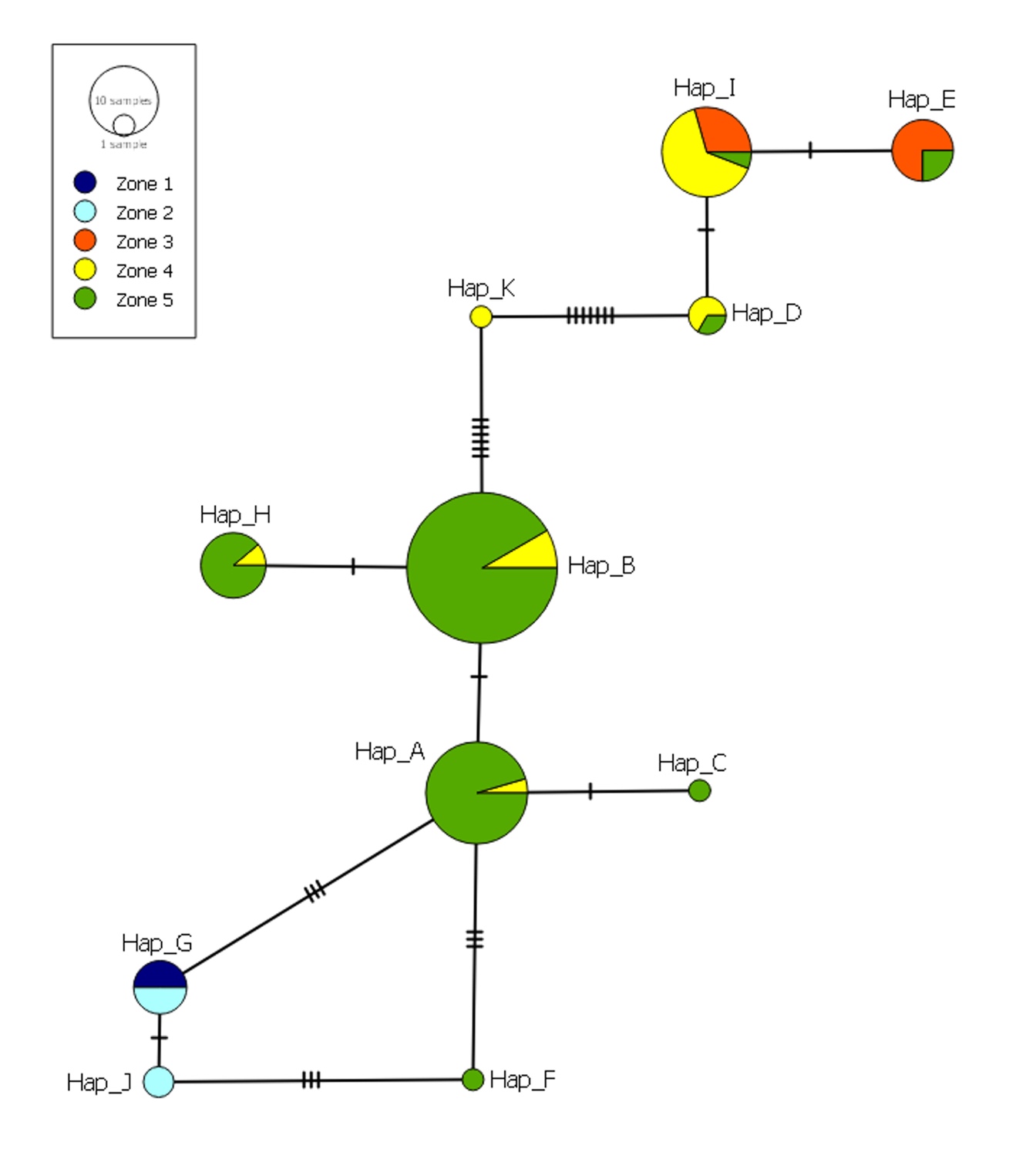


**Figure S2. Eleven median joining network haplotypes separated by regional zones.** Haplotypes G and J were found only in the Gulf of Thailand (Zones 1 and 2). In the Andaman Sea, haplotypes C, F and K were found only in Zones 4 and 5, haplotypes A, B, D and H were specific to Zones 4 and 5, haplotype E was found in Zones 3 and 5, and haplotype I was found in Zones 3, 4 and 5.
