# Supplementary material for: Genetic diversity in a unique population of dugong (Dugong dugon) along the sea coasts of Thailand
Source: Sci Rep. 2021 Jun 2;11:11624. doi: 10.1038/s41598-021-90947-4 (PMC8172547; doi:10.1038/s41598-021-90947-4)
Supplement: Supplementary file 3 — Supplementary Figure S3. [file 41598_2021_90947_MOESM3_ESM.docx]

**Genetic diversity in a unique population of dugong (*Dugong dugon*) along the sea coasts of Thailand**

Anocha Poommouang^1^, Wannapimol Kriangwanich^1^, Kittisak Buddhachat^2,3^, Janine L. Brown^4^, Promporn Piboon^1^, Siriwadee Chomdej^5^, Jatupol Kampuansai^5^, Supamit Mekchay^6^, Patcharaporn Kaewmong^7^, Kongkiat Kittiwattanawong^7^, and Korakot Nganvongpanit ^1,2*^

^1^ Department of Veterinary Biosciences and Public Health, Faculty of Veterinary Medicine, Chiang Mai University, Chiang Mai 50100, Thailand.

^2^ Excellence Center in Veterinary Bioscience, Chiang Mai 50100, Thailand.

^3^ Department of Biology, Faculty of Science, Naresuan University, Phitsanulok 65000, Thailand.

^4^ Smithsonian Conservation Biology Institute, Center for Species Survival, 1500 Remount Road, Front Royal, VA 22630, USA.

^5^ Department of Biology, Faculty of Science, Chiang Mai University, Chiang Mai 50200 Thailand

^6^ Department of Animal and Aquatic Sciences, Faculty of Agriculture, Chiang Mai University, Chiang Mai 50200, Thailand

^7^ Phuket Marine Biological Center, Phuket 83000, Thailand.

Corresponding author

Prof. Dr. Korakot Nganvongpanit

Animal Bone and Joint Research Laboratory, Department of Veterinary Biosciences and Public Health, Faculty of Veterinary Medicine, Chiang Mai University, Chiang Mai 50100, Thailand

E-mail: korakot.n@cmu.ac.th Tel.: +66-53-948046

E-mail:

AP=anopotter@hotmail.com

WK=wannapimol.k@gmail.com

KB = k_buddhachat@yahoo.com

JB= BrownJan@si.edu

PP= muy_v3@hotmail.com

SC= siriwadee@yahoo.com

JK= Jatupol_k@hotmail.com

SM= supamit.m@cmu.ac.th

PK = marineanimal.vet@gmail.com

KK = kkongkiat@gmail.com

KN = korakot.n@cmu.ac.th

**Short title:** Genetic diversity in Thailand dugong


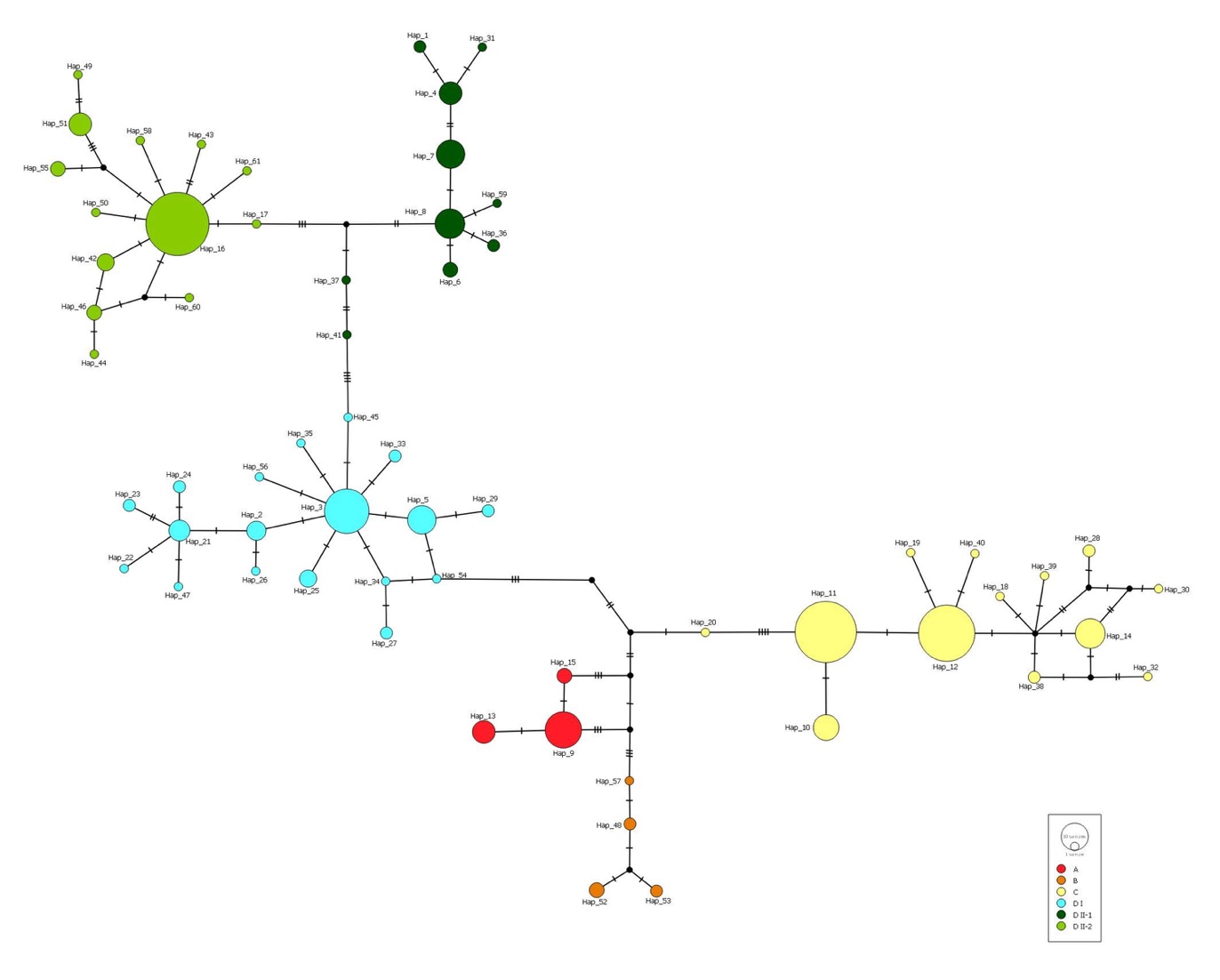


**Figure S3. Sixty-one median joining network haplotypes of global dugongs separated by clade.** Clade A (Hap9, Hap13 and Hap15), clade B (Hap48, Hap52, Hap53 and Hap57), clade C (Hap10, Hap11, Hap12, Hap14, Hap18, Hap19, Hap20, Hap28, Hap30, Hap32, Hap38, Hap39 and Hap40), clade D1 (Hap2, Hap3, Hap5, Hap21, Hap22, Hap23, Hap24, Hap25, Hap26, Hap27, Hap29, Hap33, Hap34, Hap35, Hap45, Hap47, Hap54 and Hap56), clade D2.1 (Hap1, Hap4, Hap6, Hap7, Hap8, Hap31, Hap36, Hap37, Hap41 and Hap59), clade D2.2 (Hap16, Hap17, Hap42, Hap43, Hap44, Hap46, Hap49, Hap50, Hap51, Hap55, Hap58, Hap60 and Hap61).
